# CE-QUAL-W2 model of dam outflow elevation impact on temperature, dissolved oxygen and nutrients in a reservoir

**DOI:** 10.1038/s41597-019-0316-y

**Published:** 2019-12-09

**Authors:** Karl-Erich Lindenschmidt, Meghan K. Carr, Amir Sadeghian, Luis Morales-Marin

**Affiliations:** Global Institute for Water Security, University of Saskatchewan, 11 Innovation Blvd., Saskatoon, Saskatchewan S7N 3H5 Canada

**Keywords:** Environmental impact, Limnology

## Abstract

Dams are typically designed to serve as flood protection, provide water for irrigation, human and animal consumption, and harness hydropower. Despite these benefits, dam operations can have adverse effects on in-reservoir and downstream water temperature regimes, biogeochemical cycling and aquatic ecosystems. We present a water quality dataset of water withdrawal scenarios generated after implementing the 2D hydrodynamic and water quality model, CE-QUAL-W2. The scenarios explore how six water extraction scenarios, starting at 5 m above the reservoir bottom at the dam and increasing upward at 10 m intervals to 55 m, influence water quality in Lake Diefenbaker reservoir, Saskatchewan, Canada. The model simulates daily water temperature, dissolved oxygen, total phosphorus, phosphate as phosphorus, labile phosphorus, total nitrogen, nitrate as nitrogen, labile nitrogen, and ammonium at 87 horizontal segments and at 60 water depths during the 2011–2013 period. This dataset intends to facilitate a broader investigation of in-reservoir nutrient dynamics under dam operations, and to extend the understanding of reservoir nutrient dynamics globally.

## Abstract (summary)

**Table:** 

Measurement(s)	temperature of water • concentration of oxygen in water • phosphorus • phosphate • dissolved nitrogen atom in water • nitrate • ammonium • algal production • total dissolved solids • total suspended solids
Technology Type(s)	computational modeling technique
Factor Type(s)	dam withdrawal scenarios
Sample Characteristic - Environment	dam
Sample Characteristic - Location	Lake Diefenbaker

Machine-accessible metadata file describing the reported data: 10.6084/m9.figshare.10619747

## Background & Summary

Hydropower dams are typically designed to serve as flood protection, provide water to irrigation and municipal needs, and maximize power generation. In addition, reservoirs may provide recreational activity opportunities. Despite these benefits, dam operation can also have adverse effects on in-reservoir and downstream ecosystem structure and function such as alteration of flow and water temperature regimes, biogeochemical cycling, sediment transport, and movement and fitness of biota^1,2^. Although it is widely accepted that dams have such impacts on rivers and the impoundments they create, in-reservoir impacts of dam outflow elevations on chemistry and nutrients are less studied.

Different studies have examined selective withdrawal impacts on in-reservoir and downstream temperatures^3,4^ and downstream temperature/in-reservoir dissolved oxygen (DO) tradeoffs^5^. In particular, the 2D hydrodynamic water quality CE-QUAL-W2 model^6^ has used to examine dam flow control impacts on water quality in a multi-reservoir estuarine system under an optimal channel flow scenario^7^. CE-QUAL-W2 has also been applied in some studies to examine impacts of dams and flow regulation on downstream nutrient loads, in-reservoir water quality characteristics^7–9^, and downstream outflow temperature^10^. However, to our knowledge, this model has not been used to provide insights about impacts of variable outflow elevations on in-reservoir nutrient and water chemistry dynamics.

Of special interest is Lake Diefenbaker, a large multipurpose reservoir located in the Canadian Prairies along the South Saskatchewan River (SSR). The reservoir was formed after the construction of the Gardiner and Qu’Appelle dams located on the left and right arms of the reservoir, respectively. Gardiner Dam has been managed for a combination of flood control, water supply, power generation (1,000 GWh annually), irrigation, recreation and ecological considerations, such as providing nesting habitat along the shoreline for the endangered piping plover. The reservoir not only regulates the SSR streamflows, but is also a sink for sediment and nutrients exported and transported from the SSR catchment.

Models of large reservoirs require considerable quantities of diverse input data. Data which often is not available or has low temporal and spatial resolution, make calibration difficult. Outputs of such models provide a means of examining reservoir characteristics and generate data for an open source database to facilitate further study, by-passing the redundant development and processing of similar nutrient models. The data presented here provides insights on the impacts of various withdrawal elevations on the in-reservoir nutrient and water chemistry characteristics of Lake Diefenbaker using the CE-QUAL-W2 model. The study covers the 2011–2013 period which includes a year of extreme flooding (2013) allowing inference of spillway vs turbine operation impacts on concentrations and distributions of studied variables. This data constitutes a source of information for managers to analyse and assess the effects of dam operation changes on in-reservoir dynamics and their implications on ecosystems and abstracted water quality.

## Methods

### Study site

**Fig. 1:**
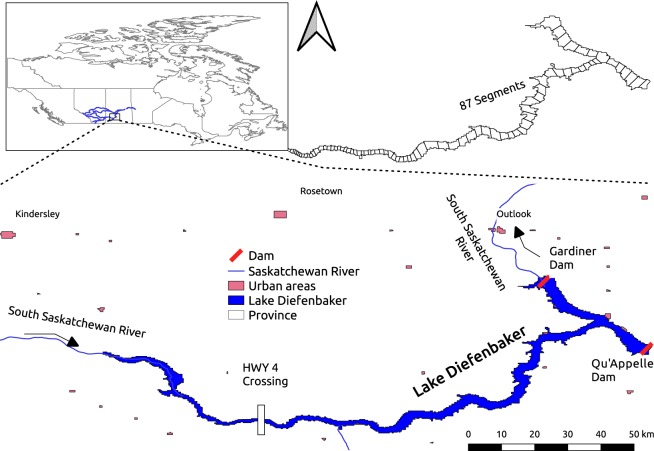
Lake Diefenbaker study area. Lake Diefenbaker, Saskatchewan, Canada. Inset shows the reservoir model segmentation into 87 longitudinal compartments. Model boundaries from the upstream HWY 4 crossing down to Gardiner and Qu’Appelle Dams are shown.

Lake Diefenbaker is a long (181.6 km) and narrow (maximum width 6 km) reservoir with a surface elevation of ~556.87 meters above sea level (full supply level) (see Fig. 1). It has a maximum depth of 60 m and a surface area of approximately 393 km^2^ with a volume of 9.03 km^3^ and a mean inflow rate of 254 m^3^/s; the reservoir has an average residence time of 1.2 years^7^. 95% of the reservoir inflows stem from the SSR and 5% from Swift Current Creek and other small tributaries. The reservoir not only serves for hydropower generation but also provides water for agricultural irrigation and domestic and industrial uses, and functions as a significant downstream flood protection measure. Since its construction, the reservoir has also been used for aquaculture and recreational activities, and serves as habitat for many aquatic animals and birds. The model simulations described here correspond to the entire length of Lake Diefenbaker extending from the upstream model boundary conditions up to the Gardiner Dam (See Fig. 1).

### Description of CE-QUAL-W2

**Fig. 2:**
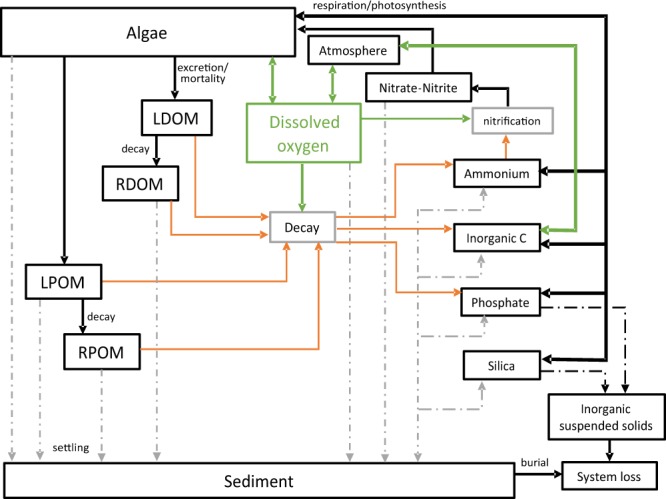
Simplified CE-QUAL-W2 model workflow diagram. Diagram of components and interactions within the CE-QUAL-W2 model based on the CE-QUAL-W2 manual^6^. LDOM, RDOM, LPOM, RPOM are labile/refractory dissolved/particulate organic matter.

CE-QUAL-W2 is a laterally-averaged water-quality and hydrodynamic model recommended by the United States Environmental Protection Agency (EPA) for comprehensive two-dimensional water quality studies. The model was selected based on the complex geometry of the reservoir and duration of the desired study period^6^. In *CE-QUAL-W2*, the laterally-averaged three-dimensional continuity and momentum (conservation of the fluid mass and conservation of momentum, respectively) equations that govern reservoir hydrodynamics are resolved numerically using finite difference methods. Furthermore, the model solves the two-dimensional advection-diffusion equation for water temperature and other water quality parameters such as suspended solids, nutrients, biological oxygen demand and algal dynamics. In order to control the model’s numerical stability, the model allows for the use of dynamic time steps where regions with frequent flow fluctuations can temporarily use smaller time steps when necessary. In total, the CE-QUAL-W2 model computes 28 state variables, 23 derived water quality variables and 73 water quality fluxes^11^ (see Fig. 2).

The CE-QUAL-W2 model has been in development for decades and, as a result, has a comprehensive user manual that covers both practical and theoretical aspects of the model as well as an active user forum^11^. The source code is freely available with clear documentation that allows for the extension and application of new algorithms and formulations^6^.

### Model implementation

The model setup for this study was taken from an earlier study performed on Lake Diefenbaker^8,12,13^. The model has 87 horizontal segments (Fig. 1) starting at Saskatchewan Highway 4 at the upstream end extending to the downstream end at Gardiner Dam and the Qu’Appelle Dam, and one-meter vertical layers with a maximum of 60 layers at the deepest point near the Gardiner Dam. Each segment of the reservoir model was also characterized by its horizontal orientation and bottom friction. The withdrawal height above the reservoir bottom of this calibrated model was altered according to six extraction scenarios starting at 5 m above the reservoir dam’s bottom at 10 m intervals up to 55 m from the bottom (5, 15, 25, 35, 45, and 55 m heights and 500, 510, 520, 530, 540 and 550 m a.s.l. respectively). Model simulations were performed from 2011 to 2013, which includes an extreme flood event in June 2013 with a return period of nearly 40 years for streamflows. Increases in flood surges were thus accommodated in the dam operations.

CE-QUAL-W2 requires multiple inputs to establish the boundary conditions (inflow, outflow, non-point source flows), meteorological forcing data and model parameters. Factors such as erosion, sedimentation, point (e.g. waste water treatment plants) and nonpoint (e.g. agricultural runoff) source loadings can greatly impact nutrient concentrations of the inflow into Lake Diefenbaker via the South Saskatchewan River. Previous to model setup, a daily water quality database was developed by correlating water quality variables with date, Julian day, discharge and water temperature at hydrometric stations located at the reservoir catchment^13^. Based on this established database, time series of water discharge, water temperature and concentrations (mg/l) of total dissolved solids (TDS), inorganic suspended solids (ISS), phosphate-P (PO_4_), ammonia-N (NH_4_), nitrate-N (NO_3_), dissolved silica (DSI), labile dissolved organic matter (LDOM), labile particulate organic matter (LPOM), algae (ALG1) and dissolved oxygen (DO) were imposed at the upstream boundary at Saskatchewan Highway 4 during the 2011–2013 period.

Similarly, outflows at the Gardiner and Qu’Appelle dams were also established as downstream boundary conditions. The results from a dynamic pump algorithm for defining outflow from Gardiner Dam to maintain the reservoir water balance were also introduced in the model. Time series of meteorological forcing data such as air temperature, dew point temperature, wind speed and direction, precipitation, solar radiation and cloud cover were also introduced in the model. Additionally, dynamic topographical and vegetation shading and wind sheltering parameters were also specified in the model. Model simulations begin on April 1, 2011, with a homogeneous temperature of 4 °C for the whole reservoir after the spring turnover, and simulations end on December 31, 2013.

## Data Records

All output data and CE-QUAL-W2 code used for this study are publicly available free-of-charge from the Federated Research Data Repository (FRDR)^14^. The repository includes a brief description of data, authors, keywords, a list of folders containing all files, and a README.txt listing all folders and files with short descriptions of the data including variables and units of measurement.

The data is composed of 8 folders: six for the selective withdrawals (5, 15, 25, 35, 45 and 55), one with individual files for each output variable and each water withdrawal (All_SIM_CSV), one for the flow fields (Flow_field) and one that contains videos of different water quality variables (FlowNutrient_videos). The All_SIM_CSV folder contains 90 comma-separated values (CSV) files with time series for each output variable (15) and each water withdrawal scenario (6) for the whole computational domain. The flow field folder itself has six subfolders each for one selective withdrawal scenario (5, 15, 25, 35, 45, 55). In each folder (model runs) there are two executable files (pre-run and run), 12 input files (all the same, except for the master file [w2_con.npt] which needs the withdrawal depth to be changed), and output files. Results are all extracted and saved in MATLAB format. A README.txt file is also included that explains the content of each file in each folder and subfolder.

### Direct video links

As part of the meta data, videos of the simulations are provided showing longitudinal and vertical profiles of the DO, nutrients, and water temperature on a daily time step for three years.

## Technical Validation

### Interpretation of model results

The key variables from model results are phosphorus, nitrogen, algae, organic matter, organic matter partitioning, suspended solids, dissolved oxygen, silica, and alkalinity. Although many restoration programs try to reduce discharge of phosphorus into receiving freshwaters, strongly bound phosphorus in benthic sediments can remain in the lake for long periods of time. Thus, many programs suggest the use of total phosphorus instead of or in addition to phosphorous (PO_4_-P) for long term rehabilitation^15^. Phosphate (PO_4_) has a strong correlation with total dissolved P (TDP) therefore TDP measurements can be used instead of phosphate as phosphorus (PO_4_^3−^P). Nitrate (NO_3_ -N) plus nitrite (NO_2_-N) is used as a single state variable. There are strict drinking water guidelines for nitrate concentrations because of severe health problems associated with elevated levels of nitrate in drinking water^16^. High nitrate concentrations can promote eutrophication in nitrogen limited waters^17^ and increase phosphorus internal loading from anaerobic sediments^18^. Ammonium, in addition to nitrate, are the primary sources of inorganic nitrogen used in algal photosynthesis for making chlorophyll pigments^19^. Algae are a major component of every water quality model as they interact with and affect the physical, chemical and biological characteristics of waterbodies. The measurements of algal biomass are usually available in the form of chlorophyll *a* which needs to be converted into biomass^20^. Modellers apply different approaches by using static or variable stoichiometric ratios to convert chlorophyll *a* concentrations into algal biomass. Organic carbon (OC) can be divided into dissolved (DOC) and particulate (POC) fractions that need to be converted to dissolved organic matter (DOM) and particulate organic matter (POM) for use in the model. Dissolved organic matter is an oxygen consumer that decays into inorganic carbon, PO_4_, and ammonium (NH_4_). Total organic carbon (TOC) is calculated as the sum of DOC and POC and then converted to organic matter by dividing the organic carbon by the carbon to biomass ratio. DOM and POM are present in two forms: labile and refractory. Labile organic matter has a short decay rate of days to weeks while refractory organic matter has a longer decay rate of years^21^. Therefore, labile compounds are removed from the water faster than refractory portions. Total dissolved solid (TDS) concentrations affect water density and have a strong linear relationship with specific conductance^22^. The measured turbidity data collected from stations along the reservoir over the course of two months (June and July 2013) were used to validate the sediment model quantitatively. DO interacts with several other water quality variables, which represent chemical, physical and biological characteristics of a waterbody and serves as a water quality index in many regions. DO is simple to calibrate but difficult to validate due to many interacting components and parameters that affect oxygen production and consumption. Silica is an essential nutrient for the growth of favourable diatoms and in limiting conditions it is succeeded by less desirable green algae and cyanobacteria^23^. In water quality models, alkalinity and total inorganic carbon (TIC) are used to predict the pH of water which in turn affects the chemical and biological reaction rates^11^ (Cole and Wells, 2015).

### Temperature model calibration and optimization

A Monte Carlo analysis with 1,000 simulations was performed to evaluate model sensitivity to different parameters, followed by automatic optimization for model calibration. A global sensitivity analysis was implemented based on the Monte Carlo analysis where each parameter set was sampled from uniform probability distributions. In order to calibrate the model more efficiently, the coupling of a global to a local optimization method was tested, which is well suited for complex systems^24^. We used the global optimization Particle Swarm Optimization (PSO) method to find optimal regions and subsequently, the local optimization Levenberg-Marquardt (LM) method to find the optimum model parameter. The PSO + LM runs were repeated until a total number of 1,000 runs was achieved. In the PSO + LM runs, instead of continuously running from 2011 to 2012, two models were used: one from April to October 2011 and the second from April to October 2012. This allowed us to check that the reservoir’s yearly net heat budget remained balanced (equal to zero) and that no residual heat was added or removed between years. Computational time also decreased without losing accuracy following this approach. The code for PSO calculations was obtained from the MATLAB® code repository (PSO, 2013) and the code for LM was obtained from a technical report^25^.

Model calibration for water temperature was performed against time series of water temperature profiles taken at 16 locations across the reservoir. The difference between observed and simulated temperature values of each Monte Carlo simulation was calculated using the sum of squared error (SSE) as:

1 $$SSE=\sum {(O-S)}^{2}$$

where *O* is the measured value and *S* is the model output time-series for vertical temperature profiles. To calculate SSE, all the temperature measurements from all different locations and depths were sorted in one column and compared with the simulated values at exactly the same depth and location. In some cases, model performance calculations were divided into smaller groups to draw out comparisons of performance for different years and depths. The root mean square error (RMSE)

2 $$RMSE=\sqrt{\sum \frac{{(0-S)}^{2}}{n}}$$

was used instead of SSE to account for differences in the number of samples between different groups. SSE values were used to find the best parameter settings in the Monte-Carlo and PSO simulations; chi-squared was used in the LM simulations.

Acceptable model performance during model calibration were obtained with RMSE less than 2 °C for simulated water temperatures. The wind sheltering coefficient (WSC) and the solar radiation shading coefficient (SHADE), which define the percentage of the wind and solar radiation that reach the water surface after passing obstacles such as topographic barriers or vegetation along the shores, were identified as the most sensitive parameters. Optimum values of 0.85 and 0.80 were found for the WSC and SHADE coefficients, respectively, during the sensitivity analysis and calibration processes. More details of model performance during calibration and validation are provided in Sadeghian *et al*.^12^.

### Water quality model calibration

The water quality model^8^ was calibrated against field measurements at the most upstream reservoir stations located at Saskatchewan Highway 4 by using the same methodology described above and in Sadeghian *et al*. (2015). Initially, the water quality model was complemented by an upstream river section to account for reverse flow effects at the upstream end of the reservoir. This initial model configuration was able to capture nutrient fluxes and streamflow characteristics more accurately avoiding data processing and transfer errors to the reservoir model. One drawback of this initial configuration was the increased computational cost during model execution. In order to reduce computational expenditure, an independent reservoir model was set up whose input data at the reservoir inlet was introduced from the initial water quality model results. Concentrations of inorganic nutrients such as PO_4_, NH_4_, NO_3_ and dissolved silica and organic components such as algae biomass and organic matter were used directly for model calibration. Based on the availability of measured data, derived variables such as particulate organic carbon (POC), particulate organic nitrogen (PON), total nitrogen (TN) and total phosphorus (TP) were also considered in the model calibration. Table 1 contains equations for calculating the derived variables and their explanations.

**Table 1:** Derived variables in CE-QUAL-W2 model.

$${\rm{POC}}={\rm{POM}}\times {{\rm{ORG}}}_{{\rm{C}}}$$	(7)
$${\rm{PON}}={\rm{POM}}\times {{\rm{ORG}}}_{{\rm{N}}}+{\rm{Algae}}\times {{\rm{A}}}_{{\rm{N}}}$$	(8)
$${\rm{TN}}={\rm{DON}}+{\rm{PON}}+{\rm{N}}{{\rm{H}}}_{4}+{{\rm{NO}}}_{3}$$	(9)
$${\rm{TP}}={\rm{DOP}}+{\rm{POP}}+{{\rm{PO}}}_{4}+{{\rm{TP}}}_{{\rm{SS}}}$$	(10)
Algae =	Algal biomass
POC =	Particulate organic carbon
PON =	Particulate organic nitrogen
POP =	Particulate organic phosphorus
TN =	Total nitrogen
TP =	Total phosphorus
POM =	Particulate organic matter
ORG_C_ =	Stoichiometric equivalent between organic matter and carbon = 0.45
ORG_N_ =	Stoichiometric equivalent between organic matter and nitrogen = 0.08
A_N_ =	Stoichiometric equivalent between algal biomass and nitrogen = 0.08
DON =	Dissolved organic nitrogen
DOP =	Dissolved organic phosphorus
NH_4_ =	Ammonium
NO_3_ =	Nitrate
PO_4_ =	Ortho-phosphate
TP_SS_ =	SS × PART_P_
SS =	suspended solids
PART_P_ =	phosphorus partitioning coefficient for suspended solids

In contrast to the calibration of the water temperature, for which a Monte-Carlo approach was implemented, the calibration of the water quality model was performed manually due to the extensive computational time. Hence, the model parameters affecting chemical and biological reactions in the system such as algal rates (growth, respiration, excretion, mortality, half-saturation for nutrients, and light saturation), algal temperature rates, algal stoichiometry, organic matter (dissolved and particulate) decay and settling rates, inorganic phosphorus sediment release rate and partitioning coefficient for suspended solids, ammonium decay rate and sediment release rate, nitrate decay rate and denitrification rate from sediments, and sediment oxygen demand were adjusted manually in the model. Such adjustment was performed by updating the parameter values after interpretation of the results after each model run, and the simulation was repeated until the required accuracy was obtained. The optimum values of the parameters determined through the calibration are provided in Sadeghian *et al*.^8^.

Model performance was evaluated based on the averaged root mean squared error (RMSEav), which is equivalent to RMSE (Equation 3) divided by the average of observed values. Values of RMSEav equal to 0.17, 0.66, 0.69, 0.38 and 0.67 were obtained for DO, POC, PON, TN and TP, respectively. According to Sadeghian *et al*.^8^, much smaller simulation errors are obtained when water samples are collected at more locations and with higher frequencies, at least weekly/monthly time intervals, for chemistry analysis. A more comprehensive description of the calibration of the water quality model and its performance is included in Sadeghian *et al*.^8^.

## Data Availability

The code used for this study is publically available at a Federated Research Data Repository (FRDR)^14^. No restrictions apply in accessing the code.
